# Characterization of a pericentric inversion in plateau fence lizards (*Sceloporus tristichus*): evidence from chromosome-scale genomes

**DOI:** 10.1093/g3journal/jkab036

**Published:** 2021-02-05

**Authors:** Ana M Bedoya, Adam D Leaché

**Affiliations:** 1 Department of Biology, University of Washington, Seattle, WA 98105, USA; 2 Burke Museum of Natural History and Culture, Seattle, WA 98105, USA

**Keywords:** chromosome rearrangement, ddRADseq, genome sequencing, hybrid zone, pericentric inversion, *Sceloporus*

## Abstract

Spiny lizards in the genus *Sceloporus* are a model system among squamate reptiles for studies of chromosomal evolution. While most pleurodont iguanians retain an ancestral karyotype formula of 2*n* = 36 chromosomes, *Sceloporus* exhibits substantial karyotype variation ranging from 2*n* =  22 to 46 chromosomes. We present two annotated chromosome-scale genome assemblies for the Plateau Fence Lizard (*Sceloporus tristichus*) to facilitate research on the role of pericentric inversion polymorphisms on adaptation and speciation. Based on previous karyotype work using conventional staining, the *S. tristichus* genome is characterized as 2*n* =  22 with six pairs of macrochromosomes and five pairs of microchromosomes and a pericentric inversion polymorphism on chromosome 7 that is geographically variable. We provide annotated, chromosome-scale genomes for two lizards located at opposite ends of a dynamic hybrid zone that are each fixed for different inversion polymorphisms. The assembled genomes are 1.84–1.87 Gb (1.72 Gb for scaffolds mapping to chromosomes) with a scaffold N50 of 267.5 Mb. Functional annotation of the genomes resulted in ∼15*K* predicted gene models. Our assemblies confirmed the presence of a 4.62-Mb pericentric inversion on chromosome 7, which contains 62 annotated coding genes with known functions. In addition, we collected population genomics data using double digest RAD-sequencing for 44 *S. tristichus* to estimate population structure and phylogeny across the Colorado Plateau. These new genomic resources provide opportunities to perform genomic scans and investigate the formation and spread of pericentric inversions in a naturally occurring hybrid zone.

## Introduction

Chromosomal rearrangements play important roles in adaptation, divergence, and speciation (Wellenreuther and Bernatchez 2018). Pericentric chromosome inversions, which are classified as inversions that include a centromere, impose significant evolutionary and ecological constraints on genome evolution by trapping alleles on inverted chromosome segments. This greatly reduces recombination with noninverted regions and increases linkage disequilibrium within inverted regions (Kirkpatrick 2010). Furthermore, when locally adapted alleles are located inside of an inversion, such as those that are ecologically relevant, the inversion can spread to fixation in a population (Kirkpatrick and Barton 2006).

The phrynosomatid lizard genus *Sceloporus* is a diverse clade containing 108 species with a broad distribution across North America (Leaché *et al.* 2016). Differentiation in the fundamental number of chromosomes is hypothesized to be a factor responsible for driving the rapid diversification of the genus (Hall 2009; Leaché and Sites Jr. 2009). While many species have the ancestral karyotype formula of 2*n* =  36 chromosomes, *Sceloporus* exhibits substantial karyotype variation (ranging from 2*n* =  22 to 46), sex chromosome evolution (Lisachov *et al.* 2020), and large genome rearrangements (Leaché and Sites Jr. 2009). Existing genomic resources for *Sceloporus* include an annotated genome for *S. undulatus* (Westfall *et al.* 2020), a *de novo* assembled shotgun genome for *S. occidentalis* and partial genomes for 34 other species (Arthofer *et al*. 2015).

Here, we present two annotated chromosome-scale genome assemblies for the Plateau Fence Lizard (*Sceloporus tristichus*), which is 2*n* = 22 with six pairs of macrochromosomes and five pairs of microchromosomes, a karyotype formula shared with all 10 member of the *undulatus* species group (Leaché *et al.* 2016). An early study of the karyological differences within the group using conventional staining techniques identified a distinctive pericentric inversion polymorphism on chromosome 7 that varies geographically (Cole 1972). This inversion polymorphism produces distinctly recognizable variants classified by the position of the centromere (Cole 1972), and individuals with heteromorphic pairs of distinct chromosome 7 inversions can be found in a dynamic hybrid zone (Leaché and Cole 2007). The hybrid zone occurs in Arizona’s Colorado Plateau at the ecotone between Great Basin Conifer Woodland and Grassland habitats (Leaché and Cole 2007). Temporal comparisons of clinal variation in kayotypes, morphology, mitochondrial DNA, and SNPs suggests that the hybrid zone is moving, possibly as a response to habitat modification (Leaché and Cole 2007; Leaché *et al.* 2017). The new genomic resources, reported here, provide a framework for increasing our understanding of the formation and spread of large chromosome inversions across broad temporal and spatial scales.

## Materials and methods

### Sampling, library preparation, and sequencing

We sequenced two female specimens of *S. tristichus* collected from a hybrid zone in Navajo County, Arizona, USA in 2002. The two individuals were karyotyped to determine the position of the centromere on chromosome 7 in a previous study (Leaché and Cole 2007). One specimen (NCBI BioSample ID SAMN15450893; voucher specimen AMNH 153954) is from the northern end of the hybrid zone (Holbrook; HOL), and is submetacentric at chromosome 7 (a nonmedial centromere that is closer to the middle than to either end of the chromosome). The second specimen (NCBI BioSample ID SAMN15450894; voucher specimen AMNH 154032) is from the southern end of the hybrid zone (Snowflake; SNOW), and is telocentric at chromosome 7 (a terminal centromere that is close to the end of the chromosome) (Table S1).

Genome sequencing was performed by Dovetail Genomics (Santa Barbara, CA, USA). High molecular weight genomic DNA was extracted from flash frozen liver tissues and validated for large fragment sizes (50–100 kb) using pulsed field gel electrophoresis. 10X Chromium libraries were prepared using the Chromium Genome Reagent Kit (10x Genomics, Pleasanton, CA, USA) followed by Illumina HiSeqX sequencing (150-bp paired-end reads). One Chicago (Putnam *et al.* 2016) and one Hi-C library (Belton *et al.* 2012) were prepared for each sample. Quality control for these libraries was performed by mapping reads (75-bp paired-end MiSeq reads) to draft 10x Supernova assemblies. Finally, both libraries were sequenced on an Illumina HiSeqX (150-bp paired-end reads). Raw sequencing reads are deposited in the NCBI Sequence Read Archive (BioProjectID PRJNA644186).

### Genome assembly and annotation

For each individual, a draft genome was *de novo* assembled from 709.35 million 10X Chromium sequence reads using the *SuperNova* assembly pipeline. Chromosome-scale scaffolding was achieved by mapping the Chicago and Dovetail Hi-C libraries back to the 10x Supernova assembly (Weisenfeld *et al.* 2017) using the *HiRise* software pipeline (Putnam *et al.* 2016).

Functional annotation of the two genomes was conducted with Funannotate v1.7.2. (Palmer and Stajich 2019) on a 64-core Ubuntu machine and the Hyak NextGen cluster supercomputer at the University of Washington. Briefly, Funnanotate aligns raw RNASeq reads to a genome sequence with minimap2 and assembles them using Trinity Grabherr *et al.* (2011). Such assemblies are used along with PASA predictions Haas *et al.* (2003) to build consensus gene model predictions, and train Augustus Stanke *et al.* (2004) and GeneMark-ES/ET Lomsadze *et al.* (2005). Repetitive regions are softmasked in Funannotate and the softmasked genomes are passed to the *ab initio* predictors. We used the Tetrapoda BUSCO (Benchmarking Universal Single-Copy Orthologs) dataset for gene prediction (Simão *et al.* 2015) and specified the option –repeats2evm, which passes repeat information to Evidence Modeler. Finally, Funnanotate performs genome functional annotations using the outputs of PFAM (Finn *et al.* 2016), MEROPS (Rawlings *et al.*, 2018), InterProScan5 (Jones *et al.* 2014), eggNOG-mapper (Huerta-Cepas *et al.* 2017), and Phobius (Käll *et al.* 2004). We ran the pipeline for each of the two *HiRise* genome assemblies and included a raw RNASeq dataset prepared from skeletal muscle of an adult female of *S. undulatus* (BioSample SAMN06312743) to build consensus gene model predictions. Functional annotation was performed on the 11 scaffolds or putative chromosomes, which included 86.4% and 91.3% of the total length of the *HiRise* assemblies (Table 1).

**Table 1:** Summary statistics for the *HiRise* genome assemblies

Metric	HOL	SNOW
Assembly size	1,870.61	1,844.30
Scaffolds >10 kb	25,615.68X	32,060.90X
L50	3	3
N50	269.061	267.474
L90	15	10
N90	17.367	36.595
Chromosome 1	366.851	366.429
Chromosome 2	304.053	319.091
Chromosome 3	269.060	267.474
Chromosome 4	249.456	247.712
Chromosome 5	173.679	170.938
Chromosome 6	164.133	162.452
Chromosome 7	50.424	50.665
Chromosome 8	38.601	38.926
Chromosome 9	36.602	36.595
Chromosome 10	19.413	16.366
Chromosome 11	11.809	11.585

Sequence lengths are given in megabases. L50, minimum number of scaffolds that make up 50% of the total assembly length; N50, minimum scaffold size of fragments that make up 50% of the total assembly length; L90, minimum number of scaffolds that make up 90% of the total assembly length; N90, minimum scaffold size of fragments that make up 90% of the total assembly length.

### Pericentric inversion polymorphism

To identify the locations of inversions on chromosome 7 between HOL and SNOW we used progressive Mauve v.2.4.0 (Darling *et al.* 2004). The program was run with default “seed families” and default values for all other parameters. We determined the identity of large scaffolds by comparing *S. tristichus* assemblies to the assembled chromosome-scale genome of the closely related species *S. undulatus* (Westfall *et al.* 2020) (Table S3 and S4). Furthermore, HiCUP v0.8.0 (Wingett et al. 2015) was used to map the Hi-C paired end raw sequencing reads of HOL against the assembled chromosome 7 of SNOW using bowtie2 v.2.4.2. Briefly, HiCUP was used to digest the SNOW genome with *DpnII*, truncate the HOL raw reads at the putative Hi-C ligation junction, map Hi-C raw reads to the SNOW digested reference genome, and remove Hi-C artefacts and putative PCR duplicates. A pairwise interaction matrix was computed using 500-kb windows with HOMER (Heinz *et al.* 2010). The matrix was normalized for sequence depth. Finally, a normalized linkage density heatmap of the first 25 Mb was generated with TreeView3 (Page 1996).

### Population genomics of the plateau fence lizard

To investigate the population structure of *S. tristichus*, we collected ddRADseq data for 44 samples from across the Colorado Plateau, USA (Table S5). The demultiplexed data are available at the NCBI Sequence Read Archive (SAMN14488621–SAMN14488665). The SNOW genome presented here was used as a reference to guide the assembly and alignment. To estimate the number of populations (*K*), we used PCA and discriminant analysis of principal components (DAPCs). A concatenated phylogenetic analysis of the SNP data using maximum likelihood with an acquisition correction model was estimated using one sample of *Sceloporus cowlesi* to root the phylogenetic tree. Detailed methods for sequencing and analysis of the population genomics data are provided in the Supplementary Materials.

### Data availability

The raw data for genome sequencing underlying this article are available at the NCBI Sequence Read Archive (SRA) under Bioproject ID: PRJNA616379 (SRR12147734–SRR12147739). GenBank accession numbers for the two genome assemblies are JACSCI000000000 (HOL) and JACSCJ000000000 (SNOW). ddRADseq raw reads are under NCBI SRA accessions (SAMN14488621–SAMN14488665). Supplementary data including annotations and Supplemental Methods, Tables S1–S8, and Figures S1–S10 are available at figshare: https://doi.org/10.6084/m9.figshare.13180268.

## Results and discussion

### Assembly and annotation

10X SuperNova assemblies included 31,453 and 30,454 scaffolds at 53.06X and 53.88X coverage, with a total assembly length of 1870.17 and 1843.78 Mb. In turn, the final *HiRise* assemblies resulted in 27,095 and 25,281 scaffolds at 25,615X and 32,060X coverage, adding to a total length of 1,870.61 and 1,844.30 Mb for HOL and SNOW, respectively. Genome assembly statistics are provided in Table 1 and in the Supplementary Material (Figures S1 and S2; Table S2 and S3). The top 11 scaffolds account for 86.44% and 91.79% of the total length of the HOL and SNOW assemblies, and comparisons to the *S. undulatus* genome confirmed that these scaffolds are the 11 chromosomes (Supplementary Figures S3 and S4). The top six scaffolds are on average ∼206 Mb longer than the subsequent five, which is expected given that *S. tristichus* has 6 macrochromsomes.

Functional annotation of the genomes resulted in up to 14,128 and 15,469 predicted gene models for HOL and SNOW, respectively (https://doi.org/10.6084/m9.figshare.13180268). Chromosome 7 spanned 864 annotated genes for HOL, whereas 1,030 were identified for SNOW. The higher number of annotated genes in SNOW is likely the result of a higher quality and more complete assembly compared to HOL as interpreted from the assembly summary statistics in Table 1.

### Pericentric inversion polymorphism

**Figure 1:**
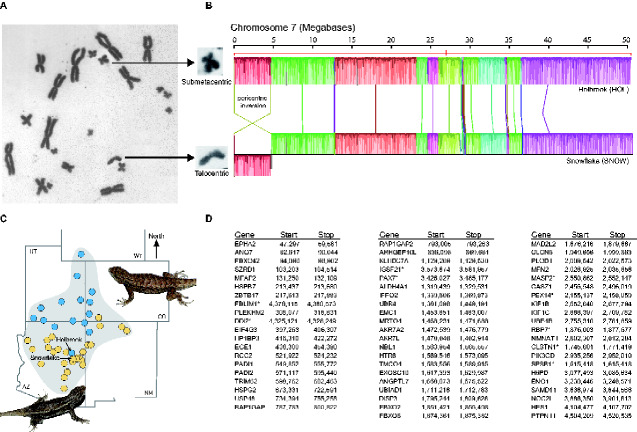
Pericentric inversion polymorphism on chromosome 7 in *Sceloporus tristichus*. (A) Karyotype of a naturally occurring hybrid from Arizona with a heteromorphic pair of chromosome 7 (telocentric + submetacentric; specimen voucher AMNH 112492; photo courtesy of Charles J. Cole). (B) Chromosome 7 alignment using Mauve for the two *S. tristichus* genomes sequenced in this study. The samples used for genome sequencing are from opposite sides of a hybrid zone (Holbrook and Snowflake). The two scaffolds that span chromosome 7 in HOL are indicated with a red line. Color bars indicate syntenic blocks, and connecting lines indicate correspondence of blocks across genomes. Blocks below the center line indicate regions that are inverted. The height of the bars indicates the average level of conservation in that region of the genome. (C) Population structure results using ddRADseq data for 44 samples assuming a *K* = 2 model supports northern and southern populations that come into close geographic proximity at a hybrid zone in Arizona between Holbrook (northern population) and Snowflake (southern population). (D) Annotated genes located inside the pericentric inversion. Genes marked with an asterisk were not annotated for HOL so their position in SNOW is given.

**Figure 2:**
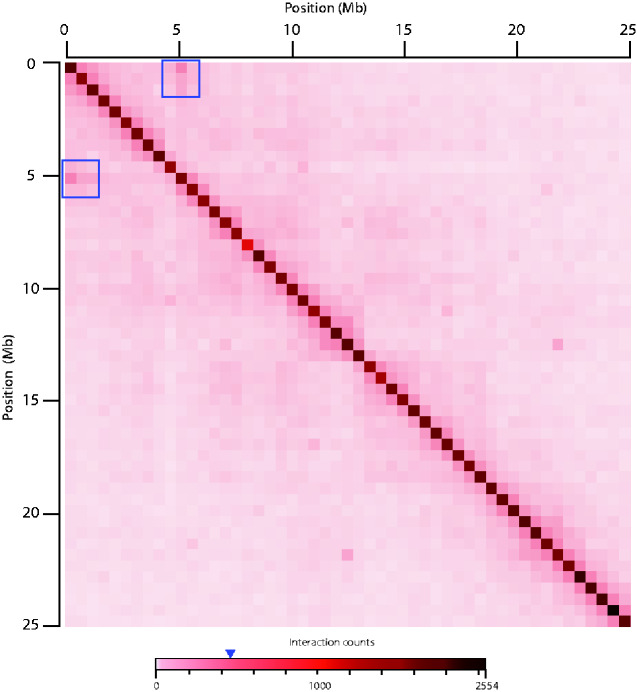
Normalized linkage density heatmap of the first 25 Mb of chromosome 7 of *S. tristichus*. Bins of 500 kb were used. The heatmap is visualized as log2 ratios of observed interactions relative to expected interactions. Raw Hi-C sequence reads of HOL were mapped to SNOW. Blue squares indicate high interactions that are visible at the length reported for the inversion in this study (4.62MB). Interactions along the diagonal are the highest, as expected. The blue arrow indicates the approximate number of interaction counts at the inversion.

Alignment of chromosome 7 with Mauve and HOL Hi-C reads mapping to chromosome 7 in SNOW resulted in the detection of a 4.62-Mb pericentric inversion (Figures 1 and 2). A chromosomal rearrangement at chromosome 7 distinguishes populations of *S. tristichus* located at opposite ends of the hybrid zone and is expected to reduce recombination in hybrids with heteromorphic pairs of chromosome 7, which are found at the center of the hybrid zone.

A total of 62 annotated genes with known function are found within the region that spans the inversion (Figure 1 and Supplementary Material Table S4). Whether any of these genes are involved in local adaptation, functional enrichment, or fixation of the inversion remains to be examined. The large size of this inversion makes it more likely that it would span multiple loci involved in local adaptation. However, it also makes it more likely to suffer meiotic costs that could reduce hybrid fitness (Kirkpatrick and Barton 2006). Adding sequencing data to refine our assemblies would help to confirm the extent of the inversion, as chromosome 7 in HOL included two scaffolds. As we used *S. undulatus* as a reference to confirm our chromosome-scale assemblies, we expect to find 1:1 synteny with *S. undulatus* (Westfall *et al.* 2020). Therefore, the pericentric inversion could be much larger than reported here, since it is possible that either one or both of the chromosome 7 scaffolds in HOL are reversed.

### Population genomics of the plateau fence lizard

Reference-based assembly of the ddRADseq data for 44 *S. tristichus* provided 8,652 loci mapping to scaffolds and a total of 20,299 SNPs (Supplementary Table S7). PCA analysis revealed regional population structuring across the Colorado Plateau, and DAPC results provide biologically plausible models for *K *=* *2, 3, and 4 (Supplementary Figures S5–S9). The hybrid zone samples (HOL and SNOW) belong to distinct populations under each of these models; HOL is a member of a northern population that extends north across the Colorado Plateau into Utah, Colorado, and Wyoming, while SNOW clusters with other southern samples from the Mogollon Rim and central Arizona. The phylogenetic analysis used 8,130 biallelic SNPs and supported clades that are concordant with the populations identified using PCA and DAPC (Supplementary Figure S10 and Supplementary Table S8). This analysis provides an evolutionary context for studying the formation of the hybrid zone, which appears to be the result of secondary contact and not primary divergence (Supplementary Figure S10).

These new genomic resources provide a starting point for understanding how a pericentric inversion polymorphism could cycle in frequency in a dynamic hybrid zone that is moving through time. Identifying the location, size, and gene content of this inversion polymorphism provides the necessary tools for investigating the role of inversions on local adaptation and speciation.

## Supplementary Material

jkab036_Supplementary_Data

## References

[jkab036-B1] Arthofer W , Banbury BL, Carneiro M, Cicconardi F, Duda TF, et al 2015. Genomic resources notes accepted 1 August 2014–30 September 2014. Mol Ecol Resour. 15:228–229. 25424247 10.1111/1755-0998.12340

[jkab036-B2] Belton J-M , McCord RP, Gibcus JH, Naumova N, Zhan Y, et al 2012. Hi–c: a comprehensive technique to capture the conformation of genomes. Methods 58:268–276. 22652625 10.1016/j.ymeth.2012.05.001 PMC3874846

[jkab036-B3] Cole CJ. 1972. Chromosome variation in North American fence lizards (genus *Sceloporus*; *undulatus* species group). Syst Biol. 21:357–363.

[jkab036-B4] Darling AC , Mau B, Blattner FR, Perna NT. 2004. Mauve: multiple alignment of conserved genomic sequence with rearrangements. Genome Res. 14:1394–1403. 15231754 10.1101/gr.2289704 PMC442156

[jkab036-B5] Finn RD , Coggill P, Eberhardt RY, Eddy SR, Mistry J, et al 2016. The pfam protein families database: towards a more sustainable future. Nucleic Acids Res. 44:D279–D285. 26673716 10.1093/nar/gkv1344 PMC4702930

[jkab036-B6] Grabherr MG , Haas BJ, Yassour M, Levin JZ, Thompson DA, et al 2011. Trinity: reconstructing a full-length transcriptome without a genome from RNA-Seq data. Nat Biotechnol. 29:644–652. 21572440 10.1038/nbt.1883 PMC3571712

[jkab036-B7] Haas BJ , Delcher AL, Mount SM, Wortman JR, Smith RK Jr., et al 2003. Improving the Arabidopsis genome annotation using maximal transcript alignment assemblies. Nucleic Acids Res. 31:5654–5666. 14500829 10.1093/nar/gkg770 PMC206470

[jkab036-B8] Hall WP. 2009. Chromosome variation, genomics, speciation and evolution in *Sceloporus* lizards. Cytogenet Genome Res. 127:143–165. 20339293 10.1159/000304050

[jkab036-B9] Heinz S , Benner C, Spann N, Bertolino E, Lin YC, et al 2010. Simple combinations of lineage-determining transcription factors prime cis-regulatory elements required for macrophage and b cell identities. Mol Cell 38:576–589. 20513432 10.1016/j.molcel.2010.05.004 PMC2898526

[jkab036-B10] Huerta-Cepas J , Forslund K, Coelho LP, Szklarczyk D, Jensen LJ, et al 2017. Fast genome-wide functional annotation through orthology assignment by eggNOG-mapper. Mol Biol Evol. 34:2115–2122. 28460117 10.1093/molbev/msx148 PMC5850834

[jkab036-B11] Jones P , Binns D, Chang H-Y, Fraser M, Li W, et al 2014. InterProScan 5: genome-scale protein function classification. Bioinformatics 30:1236–1240. 24451626 10.1093/bioinformatics/btu031 PMC3998142

[jkab036-B12] Käll L , Krogh A, Sonnhammer EL. 2004. A combined transmembrane topology and signal peptide prediction method. J Mol Biol. 338:1027–1036. 15111065 10.1016/j.jmb.2004.03.016

[jkab036-B13] Kirkpatrick M. 2010. How and why chromosome inversions evolve. PLoS Biol. 8:e1000501. 20927412 10.1371/journal.pbio.1000501 PMC2946949

[jkab036-B14] Kirkpatrick M , Barton N. 2006. Chromosome inversions, local adaptation and speciation. Genetics 173:419–434. 16204214 10.1534/genetics.105.047985 PMC1461441

[jkab036-B15] Leaché AD , Banbury BL, Linkem CW, Nieto-Montes de Oca A. 2016. Phylogenomics of a rapid radiation: is chromosomal evolution linked to increased diversification in North American spiny lizards (Genus *Sceloporus*)? BMC Evol Biol. 16:63. 27000803 10.1186/s12862-016-0628-x PMC4802581

[jkab036-B16] Leaché AD , Cole CJ. 2007. Hybridization between multiple fence lizard lineages in an ecotone: locally discordant variation in mitochondrial DNA, chromosomes, and morphology. Mol Ecol. 16:1035–1054. 17305859 10.1111/j.1365-294X.2006.03194.x

[jkab036-B17] Leaché AD , Grummer JA, Harris RB, Breckheimer I. 2017. Evidence for concerted movement of nuclear and mitochondrial clines in a lizard hybrid zone. Mol Ecol. 26:2306–2316. 28133829 10.1111/mec.14033

[jkab036-B18] Leaché AD , Sites J. Jr. 2009. Chromosome evolution and diversification in North American spiny lizards (genus *Sceloporus*). Cytogenet Genome Res. 127:166–181. 20203475 10.1159/000293285

[jkab036-B19] Lisachov A , Tishakova K, Romanenko S, Molodtseva A, Prokopov D, et al 2020. Whole-chromosome fusions in the karyotype evolution of *Sceloporus* (Iguania, Reptilia) are more intense in sex chromosomes than autosomes. bioRxiv. [10.1101/2020.03.31.011619] 10.1098/rstb.2020.0099 PMC8310709 34304596

[jkab036-B20] Lomsadze A , Ter-Hovhannisyan V, Chernoff YO, Borodovsky M. 2005. Gene identification in novel eukaryotic genomes by self-training algorithm. Nucleic Acids Res. 33:6494–6506. 16314312 10.1093/nar/gki937 PMC1298918

[jkab036-B21] Page RD. 1996. Tree view: an application to display phylogenetic trees on personal computers. Bioinformatics 12:357–358. 10.1093/bioinformatics/12.4.357 8902363

[jkab036-B22] Palmer J , Stajich J. 2019. Funannotate v1.5.3. Zenodo p. 10.5281/zenodo.2604804.

[jkab036-B23] Putnam NH , O’Connell BL, Stites JC, Rice BJ, Blanchette M, et al 2016. Chromosome-scale shotgun assembly using an *in vitro* method for long-range linkage. Genome Res. 26:342–350. 26848124 10.1101/gr.193474.115 PMC4772016

[jkab036-B24] Rawlings ND , Barrett AJ, Thomas PD, Huang X, Bateman A, et al 2018. The MEROPS database of proteolytic enzymes, their substrates and inhibitors in 2017 and a comparison with peptidases in the PANTHER database. Nucleic Acids Res. 46:D624–D632. 29145643 10.1093/nar/gkx1134 PMC5753285

[jkab036-B25] Simão FA , Waterhouse RM, Ioannidis P, Kriventseva EV, Zdobnov EM. 2015. BUSCO: assessing genome assembly and annotation completeness with single-copy orthologs. Bioinformatics 31:3210–3212. 26059717 10.1093/bioinformatics/btv351

[jkab036-B26] Stanke M , Steinkamp R, Waack S, Morgenstern B. 2004. AUGUSTUS: a web server for gene finding in eukaryotes. Nucleic Acids Res. 32:W309–W312. 15215400 10.1093/nar/gkh379 PMC441517

[jkab036-B27] Weisenfeld NI , Kumar V, Shah P, Church DM, Jaffe DB. 2017. Direct determination of diploid genome sequences. Genome Res. 27:757–767. 28381613 10.1101/gr.214874.116 PMC5411770

[jkab036-B28] Wellenreuther M , Bernatchez L. 2018. Eco-evolutionary genomics of chromosomal inversions. Trends Ecol Evol. 33:427–440. 29731154 10.1016/j.tree.2018.04.002

[jkab036-B29] Westfall AK , Telemeco RS, Grizante MB, Waits DS, Clark AD, et al 2020. A chromosome-level genome assembly for the Eastern Fence Lizard (*Sceloporus undulatus*), a reptile model for physiological and evolutionary ecology. bioRxiv. [10.1101/2020.06.06.138248] 10.1093/gigascience/giab066 PMC8486681 34599334

[jkab036-B30] Wingett S , Ewels P, Furlan-Magaril M, Nagano T, Schoenfelder S, et al 2015. Hicup: pipeline for mapping and processing hi-c data. F1000Res. 4:1310. 26835000 10.12688/f1000research.7334.1 PMC4706059

